# Human gastric magnet-controlled capsule endoscopy conducted in a standing position: the phase 1 study

**DOI:** 10.1186/s12876-019-1101-2

**Published:** 2019-11-12

**Authors:** Chun-sheng Cheng, Ting-ji Sun, Hou-de Zhang

**Affiliations:** Department of Gastroenterology, Shenzhen Sixth People’s Hospital, Guangdong Medical University, Nanshan District, Shenzhen, 518052 China

**Keywords:** Fundus, Cardia, Visibility, Magnetic, Endoscope, Stomach

## Abstract

**Background:**

Current magnet-controlled capsule endoscopy (MCE) for the stomach is not yet satisfactory with respect to navigation control, especially in the gastric fundus and cardia. A newly developed MCE system conducted in a standing rather than supine position may improve capsule maneuverability within the stomach. The aim of this phase 1 study was to assess the feasibility and safety of this system for examining the human stomach in healthy volunteers.

**Methods:**

A cohort of 31 healthy volunteers were enrolled. Each swallowed a capsule after drinking water and gas producing agents intended to produce distention. Under the newly developed standing MCE system, subjects were examined endoscopically while standing with external guide magnets placed on the abdominal wall and left lower chest. Safety, gastric preparation, maneuverability, visualization of anatomical landmarks and the gastric mucosa, and examination time were the primary parameters assessed. The gastric preparation and examination procedures were well accepted by the subjects and there were no adverse events.

**Results:**

Gastric examination took 27.8 ± 8.3 min (12–45 min). Gastric cleanliness was good in 24 participants (77.4%) and moderate in 7 participants (22.6%). Gastric distention was good in all of 31 participants (100%). Capsule maneuverability was also graded as good in all 31 subjects (100%), and manipulation in the fundus and cardia regions was as easy as that in the antrum and body. Visualization of the gastric cardia, fundus, body, angulus, antrum and pylorus was assessed subjectively as complete in all 31 subjects (100%). Visualization of the gastric mucosa was also good (> 75%) in all 31 subjects (100%). In areas where the mucosa could not be visualized, the low visibility was due to opaque fluid or foam. Polyps and erosive lesions were found in 25 subjects.

**Conclusion:**

MCE of the stomach conducted in a standing position is feasible and safe with satisfactory maneuverability.

## Background

The invention of a wireless capsule endoscope by Israeli and British scientists in 2000 brought about a new era of painless minimally invasive gastrointestinal endoscopy [1–4]. However, the original capsules, which are propelled by gastrointestinal peristalsis are not suitable for a thorough examination of the wide, spacious stomach. A decade later, the same Israeli team took the lead once more, testing the first magnet-controlled capsule endoscope for use in the stomach in a young adult. Under synchronous observation of traditional upper gastrointestinal endoscopy (UGIE), it was documented that the capsule could be propelled to move rapidly and precisely to any designated location by moving a handheld external magnet around the torso of the volunteer [5]. However, attempts to replicate that study with products that were clinically approved in China or available for research were unable to recreate the flexibility of manipulation described in the first trial, regardless of whether the magnetic capsule was manipulated with a handheld magnet or with a robot-assisted magnetic manipulation system. Furthermore, operators exploring the stomach encounter blind areas, especially in the gastric fundus and cardia [6–14].

Based on radiological and endoscopy modeling, we speculate that the exciting observation of flexibility in first case report was due to the continuous gas injection at the time of synchronous UGIE, which maintains persistent dilatation with diminishment of the angle between the longitudinal axis of the gastric fundus and the longitudinal axis of the gastric body as well as flattening of coarse junction ridges between gastric fundus and body. Furthermore, our model analysis has also shown the optimal position for magnet-controlled capsule endoscopy (MCE) to be standing rather than supine, owing to concurrent disappearance of the hindering angle and coarse junction ridges [accepted and pending publication data]. With respect to placement of the external controlling magnet, placement on the anterior abdominal wall has been shown to be only suitable for exploring the gastric antrum and body. For exploration of the gastric fundus and cardia, the external magnet should instead be placed on left lower chest, where its vertical distance to distal points of the fundus and cardia is minimized, thereby maximizing its magnetic power at a given magnetic induction level.

Based on the results of our model analysis, a newly MCE system suitable for use in patients standing position was developed. Follow its successful trial in pig stomachs, the aim of this pilot study was to assess the feasibility and safety of this system for examination of the human stomach in healthy volunteers.

## Methods

### Subjects

From October to December 2018, 31 healthy volunteers, including 11 men and 20 women (age, range 21–65 years), were recruited to the study. They were all absent of any concerning abdominal symptoms. None had taken any medications in the prior 3 months or undergone any abdominal surgery in their lives. Written informed consent was obtained from each participant. The study protocol (registration NO.CHiCTR1800018824) was approved by Shenzhen Sixth People’s Hospital ethics committee. The study wan performed in accordance with the Declaration of Helsinki.

### Devices

**Fig. 1:**
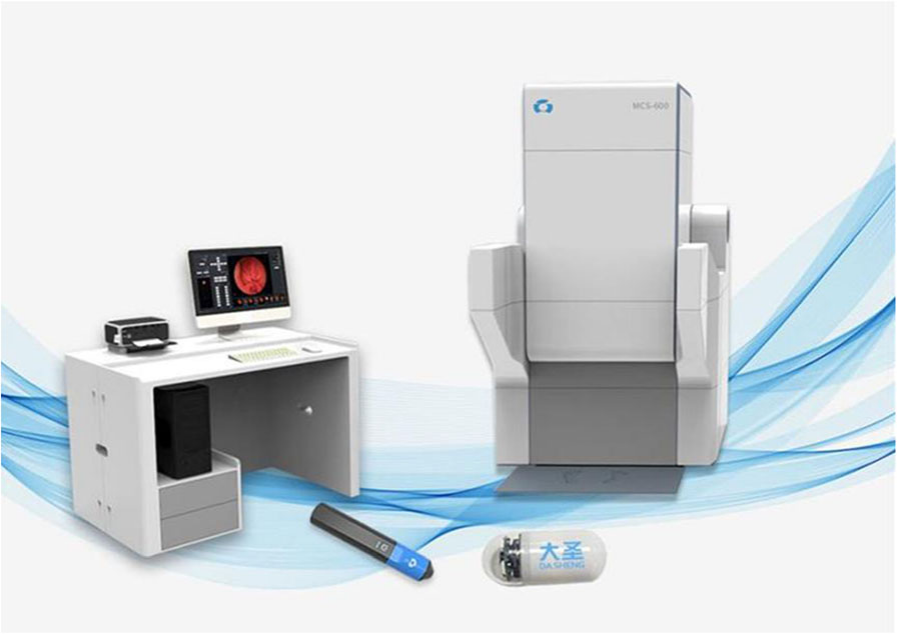
Magnet-controlled capsule endoscopy system for use in standing patients. The system consists of an ingestible capsule endoscope (foreground right), a guidance magnet robot (right), a data recorder (left foreground), and a computer workstation (left) with software for real-time viewing and control

As shown in Fig. 1, our newly developed MCE system consists of four main subsystems: an ingestible capsule endoscope; a guidance magnet robot; a data recorder; and a computer workstation with software for real-time viewing and control (all components from FUZI Technologies Co. LTD, Shenzhen, China). The capsule endoscope is a modification of a standard FUZI Dasen capsule that was approved for clinical examination of the small bowel. The magnetic control system controls the movement of the large magnet in the magnetron control equipment through the magnetron workstation equipped with control software. The large magnet can move in the plane perpendicular to the ground plane at 500x500mm, and can change its attitude at any angle through two rotating axes. Capsule endoscopy contains magnetic dipole. The magnetic control system controls the capsule endoscopy through the traction force of the large magnet to the dipole. The dipole inside the capsule interacts with the magnet outside to control the movement of the capsule, such as translation and rolling. When the big magnet moves, the capsule endoscope can move to the predetermined position together (unless the capsule is blocked by the gastric wall, then try to change the path of the magnet motion so that the capsule can bypass the obstruction). When the big magnet rotates along two rotating axes, the capsule also rotates with the rotation, taking full-angle photographs of the gastric wall in front of the lens. The capsule, which is 27 mm × 12 mm in size, has a single CMOS sensor and a permanent magnet inside its dome [Fig. 1]. (The view depth is 0-50 mm and the view angle is 136^0^ The image resolution is 480 × 480.) The capsule is activated by infrared photoelectricity rather than magnetically, as the original model had been. Its capture rate is four frames per second with a 30~40-min power supply. Under the guidance of a magnetic navigation robot, the capsule can perform six basic movements, including forward, backward, ascending, descending, rotating and flipping motions.

A data recorder with an antenna is integrated on the magnetic navigation robot. Image signals from the capsule are transmitted directly to the antenna of the data recorder, obviating the need for commonly used wire-linked skin contact sensors. The images are displayed and stored in real time on the workstation monitor, simultaneously.

The magnetic navigation robot is shaped like the backplane of the human chest X-ray machine standing upright. The manipulating magnet, which is installed behind the flat plate, can move horizontally, vertically, or in rotary motion under the control of a mechanical manipulating arm. The maximum intensity of the magnetic field generated by the magnetic robot is 200 + 50mT. The capsule can be controlled by the magnetic robot even at a distance of 300 mm.

The computer workstation provides a real-time display and control platform. Capsule movement is manipulated by mode click and a joystick. The operator conducts the examination by clicking the needed action icon. Briefly, the basic motion direction icons are presented in the computer control interface, including raise, lower, move left, move right, and rotate 180° and 360°. An icon click moves the capsule within a 3-cm range. In addition, the control interface has fine tuning icons that provide smaller movements, that is, 15° adjustments and 1 cm movements.

### Examination procedure

**Fig. 2:**
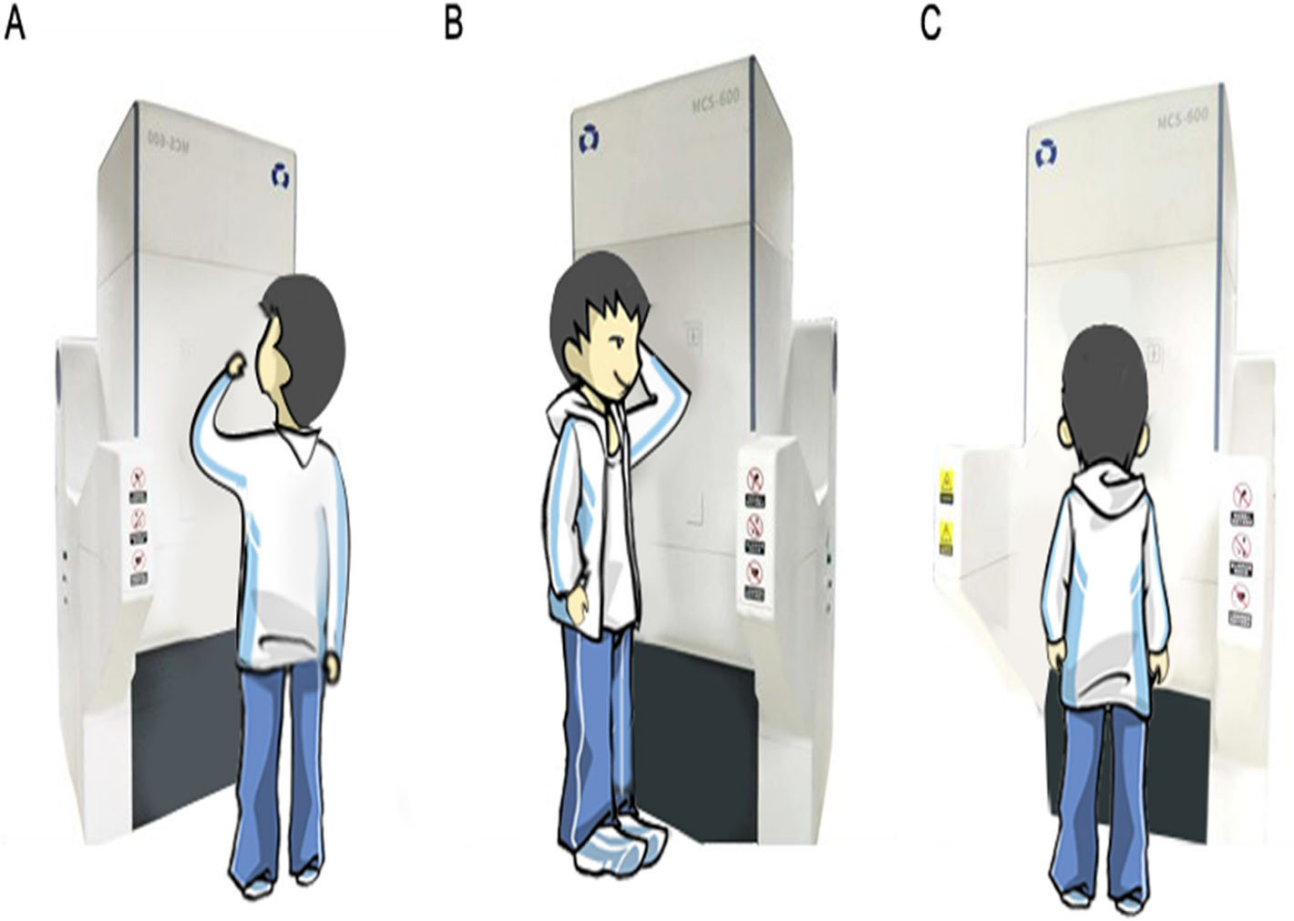
Subject positions during the examination. **a** Capsule swallowing and identification of the gastric angylus. **b** Examination of the gastric cardia and fundus. **c** Navigation of the gastric antrum and pylorus

The subjects arrived at the hospital between 8:00 am and 10:00 am after fasting overnight (> 8 h) and drinking 500 ml of warm water shortly after waking in the morning. About an hour before capsule ingestion, 10 ml of simethicone emulsion solution was administrated orally with 200 ml of clear warm water, followed by 80 ml of mucus scavenger (SZ 2000 U and 1.0 g sodium bicarbonate dissolved in 80 ml of water at 30–40 °C) about 15 min before ingestion. At the beginning of each examination, the subject was asked to stand upright with his or her chest and abdomen close to the magnetic control plate, and then to swallow the infrared activated magnet-control capsule endoscope followed by 500 ml of drinking water within 3 min [Fig. 2].

After the capsule reached the stomach, the investigator sat in front of the workstation and directed the capsule navigation by clicking on action icons. First, the investigator identified the gastric anglus, which is used as the orienting point for navigation; the capsule would be guided back to the anglus if it went off course. Second, the subjected was instructed to turn with his or her left hand on his or her head to orient the left side of the body close to the magnetic control plate, at which point the capsule was guided to turn and cruise through the cardia, fundus, greater curvature, and lesser curvature of the gastric body, and then return to the anglus. Third, the subject was instructed to return to the original position facing the magnetic control plate, after which the capsule was guided through the greater curvature and lesser curvature of the antrum and the pylorus, and then to return to the anglus. After completion of the gastric navigation course, the capsule was guided into the duodenum. If gastric distension was unsatisfactory at any time during the examination, the subject was asked to drink additional water (200 ml/time).

### Study end points

The study end points were safety, gastric preparation quality, and feasibility. Evaluations of these indexes were completed by two investigators with substantial endoscopy experience, and the lower index value of the two was selected for further analysis.

### Safety

Safety was assessed according to the occurrence of adverse events and the tolerability of subjects, as assessed by questionnaire.

### Quality of gastric preparation

Gastric preparation quality included degree of cleanliness and distention. Cleanliness was graded as good (transparent fluid and < 5% of the gastric mucosa obscured), moderate (slightly opaque fluid and/or 5–10% of the gastric mucosa obscured), or poor (opaque fluid and/or > 10% of gastric mucosa obscured. Distention was classified as good (satisfactory gastric distention with a small amount of collapsed gastric folds), moderate (considerable collapse of gastric folds obscuring limited parts of the stomach) or poor (noninflated gastric cavity was not inflated making examination of areas of interest impossible).

### Feasibility

Feasibility was evaluated in terms of three end points: maneuverability, visualization, and time. MCE maneuverability was graded as good (followed control signals reliably and moved to targeted anatomical landmarks precisely), moderate (followed control signals and moved towards the target but did not reach it precisely), or poor (failure to follow controls). Visualization of primary anatomical landmarks and the gastric mucosa were assessed. The former was expressed as the discovery rate of the cardia, fundus, body, angulus, antrum, and pylorus of stomach. The latter was graded as good (> 75% of mucosa observed), moderate (50–75% observed), or poor (< 50% observed). The total examination time recorded was the time from the swallowing of the capsule to the end of the examination with the capsule entering the descending portion of the duodenum.

### Statistical analysis

Descriptive statistics were used to describe the demographic characteristics of the participants, quality of gastric preparation, feasibility of the MCE examination, and lesion findings.

## Results

### Safety

All 31 healthy volunteers completed the examination, tolerating it well and accepting it without complaint. There were no reports of discomfort in the preparation process, nor throughout the entire examination. All 31 subjects affirmed that they would be very willing to accept the examination again if necessary. All capsules were discharged without incident within 72 h of the examination. In the week after the examination, follow-up revealed no complaints of abdominal pain, diarrhea, or other abdominal symptoms.

### Gastric preparation

Gastric cleanliness was good in 24 (77.4%) and moderate in 7 (22.6%) of the 31 subjects; there were no cases of poor cleanliness. All 31 subjects (100%) had good distention.

### Feasibility

#### Maneuverability

Under the guidance of our magnet robot, the capsules were moved smoothly to the target site with good maneuverability in all 31 subjects. Prior MCE difficulties with navigation in the cardia and fundus were not encountered in this study. Rather, the capsule was manipulated as easily in the cardia and fundus as it was in the antrum and body.

#### Duration

The duration of examination was longest in the first few cases, showing a progressive shortening as we accumulated experience. The average examination time was 27.8 ± 8.3 min (range, 12–45 min).

#### Anatomical markers and mucosa

**Fig. 3:**
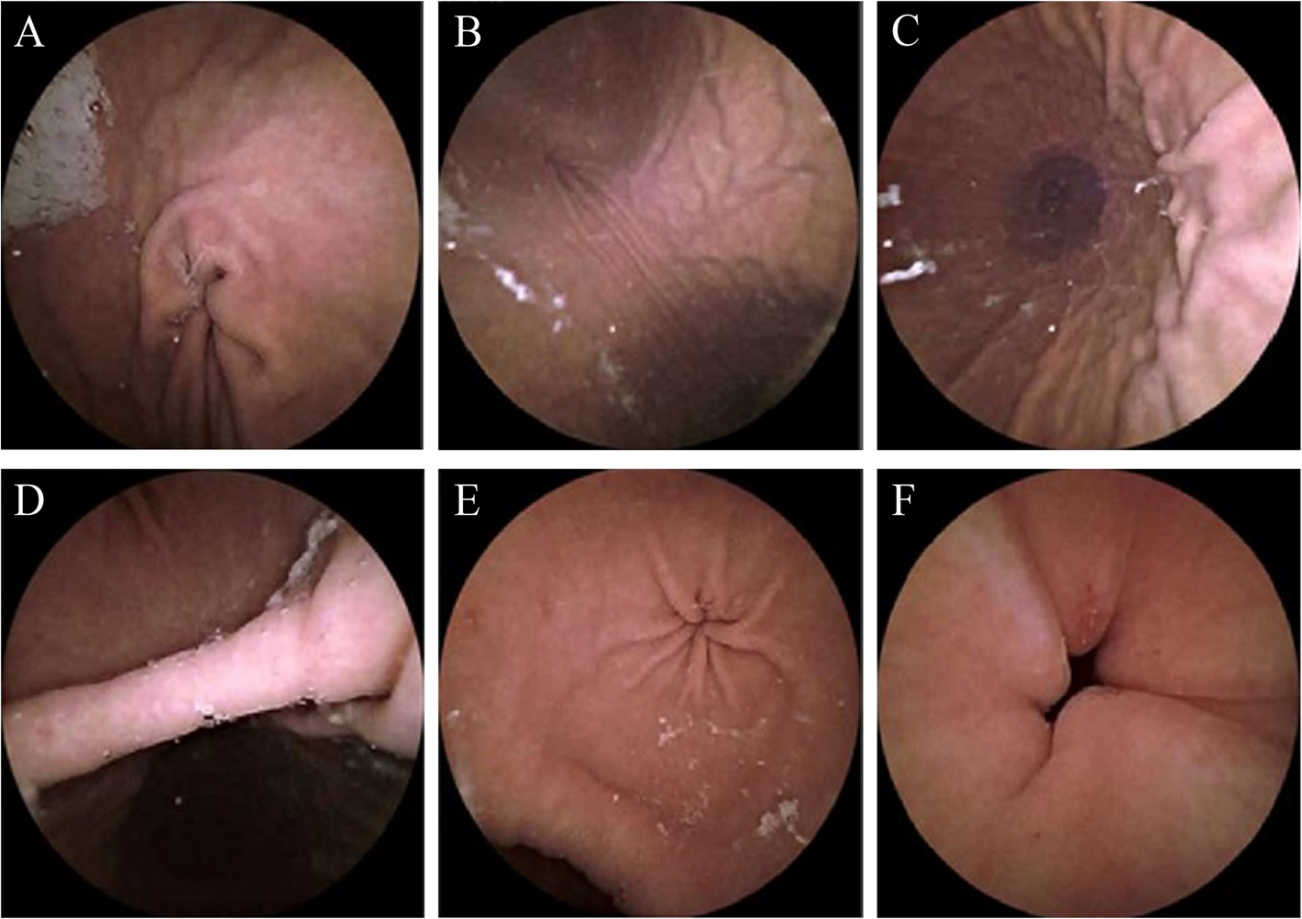
The images of primary landmarks in the stomach, namely the cardia (**a**), fundus (**b**), body (**c**), angulus (**d**), antrum (**e**), and pylorus with erosion (**f**)

As shown in Fig. 3, the anatomical landmarks of the stomach, including the gastric cardia, fundus, body, angulus, antrum, and pylorus, were well visualized in all 31 subjects (100%). Visualization of the gastric mucosa was good (> 75%) in all 31 subjects. The limited instances of mucosa visualization loss were due to opaque fluid in every case.

#### Pathological findings

The lesions were found in 7 subjects (23.3%), including 3 tiny polyps located in greater curvature in 2 subject (6.7%) and erosive lesions in 6 (20.0%). Polyps were confirmed to be fundic gland hyperplasia with biopsy pathology on the subsequent invasive gastroscopy, while 28 remainders refused further examination of free gastroscopy.

#### Imaging of the esophagus and duodenum

Capsule movement in the esophagus and duodenum was uncontrolled and passive. No whole esophageal and duodenal image series were obtained. Front-view cardia images were obtained in 5 subjects (16.1%); duodenal bulb images were obtained in 2 subjects (6.5%) (Additional file 1).

## Discussion

This study is the first reported human trial of gastric MCE conducted in a standing position. All 31 healthy volunteers completed the MCE examination safely and without difficulty. The capsule’s safety and tolerance can be attributed to the fact that it was modified from previously approved capsules, with the sealed shell material and size unchanged. A noteworthy and exciting outcome of this study was the outstanding maneuverability of the new MCE system. The operator was able to manipulate the capsule within the stomach to all selected targets easily and precisely. Navigation difficulties encountered in previous studies conducted with subjects lying in a supine position were not encountered in this study. All six anatomical landmarks of the stomach were well visualized in all 31 subjects. To the limited extend that areas of the mucosa could not be visualized, the obstacle to visualization was the presence of opaque fluid or foam in all instances, with no visualization difficulties being attributable to capsule maneuverability.

Gastrointestinal endoscopy involves three procedures: inflation to maintain the visual field; bringing the endoscope to target locations by way of advancement, retreat, and rotation. Correspondingly, MCE must meet three basic conditions: minimal obstacles to capsule movement; clear visualization during imaging; and sufficient magnetic force to bring the capsule through its gastrointestinal environment. Through radiological and endoscopic model examination of several subject positions during MCE, we found that the supine and standing positions were the least and most amenable to MCE, respectively. These modeling analyses also revealed that optimal capsule control was achieved by adding a second external magnetic body site at the left lower chest in addition to the anterior abdominal wall.

In the supine decubitus position, the stomach is horizontal relative to the body and located mainly in the left hypochondriac region, such that gravity produces a sharp acute angle between the longitudinal axis of the fundus and the longitudinal axis of the gastric body, and a rough ridge at the junction between the gastric fundus and body that impedes capsule movement substantially. Unless there is continuous injection of gas to maintain dilation, as in the first case report [5], the sharp acute angle cannot be flattened by drinking water and a gas producing agent alone due to belching and gastric emptying. The angle is less severe, but still cumbersome, in left lateral, prone, and right lateral decubitus, than in the supine position. When the subject stands upright, however, the stomach shifts to a vertical position, descending to near the ilium, approaching the pelvis. The angle becomes flattened to near 180° while the fundus’ longitudinal axis comes to nearly overlap the longitudinal body axis. The ridge between the gastric fundus and body that is present in subjects lying down is removed in standing subjects, eliminating the greatest obstacle to intragastric movement of the capsule.

The power of the external magnet to compel capsule movement is a key factor for free navigation. Because magnetic strength is related inversely to distance, magnetic power at a given induction intensity is maximized by minimizing the distance between the capsule target region and the body surface where the controlling magnet is to be placed. Because the gastric fundus is near the left lateral lower chest, we place the magnet on the left lower chest to control the capsule while it is in this area, overcoming previously described difficulties with having sufficient magnetic force during fundus/cardia exploration. In our system with a standing subject, the problem is resolved easily by having the subject rotate so that his or her left body side is closest to the magnetron.

Although excellent capsule maneuverability in MCE was demonstrated, this study had several noteworthy limitations. First, we had precise control of the capsule only within the stomach, while movements in the esophagus and duodenum were passive, yielding images purely by chance. To achieve the goal of active examination of the entire upper gastrointestinal tract, intensive further research is needed. A string-pulled capsule endoscope may be a potential solution [14]. Second, the cleanliness of the stomach was not ideal. The incomplete mucosal visualization encountered due to opaque fluid or foam is a complex issue requiring further work to resolve. During traditional UGIE, mucus, foam, and food residue can be discharged easily by suction and washing. However, such discharge remains a major challenge in capsule endoscopy.

## Conclusion

MCE conducted in a standing position had excellent maneuverability and detectability. It was accepted without complaints and well tolerated. Based on these observations, we recommend a large-scale clinical validation study of this new MCE system.

## Supplementary information

**Additional file 1**: Basic data of the volunteer examination results.

## Data Availability

The datasets used during the current study are included in the supplementary information file and also are available in ReaMan (www.medresman.org).

## Abbreviations

UGIE: Upper gastrointestinal endoscopy
MCE: Magnet-controlled capsule endoscopy
